# Portocaval shunt can optimize transhepatic flow following extended hepatectomy: a short-term study in a porcine model

**DOI:** 10.1038/s41598-022-05327-3

**Published:** 2022-01-31

**Authors:** Mohammad Golriz, Ali Majlesara, Elias Khajeh, Nahid Rezaei, Arash Saffari, Jalal Arwin, Mohammadreza Hafezi, Saroa El Sakka, Sepehr Abbasi, Golnaz Emami, Ali Ramouz, Arianeb Mehrabi

**Affiliations:** Department of General, Visceral and Transplantation Surgery, University of Heidelberg, Im Neuenheimer Feld 420, 69120 Heidelberg, Germany

**Keywords:** Cancer, Diseases, Gastroenterology

## Abstract

The aim of this study was to evaluate whether the portocaval shunt (PCS) corrects these unwanted changes in transhepatic flow after extended hepatectomy (EH). Forty female Landrace pigs were divided into two main groups: (A) EH (75%) and (B) no EH. Group A was divided into 3 subgroups: (A1) EH without PCS; (A2) EH with side-to-side PCS; and (A3) EH with end-to-side PCS. Group B was divided into 2 subgroups: (B1) side-to-side PCS and (B2) end-to-side PCS. HAF, PVF, and PVP were measured in each animal before and after the surgical procedure. EH increased the PVF/100 g (173%, p < 0.001) and PVP (68%, p < 0.001) but reduced the HAF/100 g (22%, p = 0.819). Following EH, side-to-side PCS reduced the increased PVF (78%, p < 0.001) and PVP (38%, p = 0.001). Without EH, side-to-side PCS reduced the PVF/100 g (68%, p < 0.001) and PVP (12%, p = 0.237). PVP was reduced by end-to-side PCS following EH by 48% (p < 0.001) and without EH by 21% (p = 0.075). PCS can decrease and correct the elevated PVP and PVF/100 g after EH to close to the normal values prior to resection. The decreased HAF/100 g in the remnant liver following EH is increased and corrected through PCS.

## Introduction

Liver resection is the most common and most efficient treatment for primary and secondary hepatic tumors, one that can provide a chance of long-term survival^1–3^. Improvements in patient selection criteria, surgical methods, and postoperative care have increased the indications for therapeutic extended hepatectomy (EH)^4,5^. The most important complication of EH is posthepatectomy liver failure or small for size and flow (SFSF) syndrome, which is associated with significantly high rates of morbidity and mortality^6,7^. The current therapeutic methods are not effective because most often, early tissue damage following SFSF is irreversible, and the liver parenchyma loses its capability to regenerate. Thus, the best approach is to predict the chance of SFSF and to perform the appropriate preventive procedure.

Current viewpoints strongly indicate that the incidence of SFSF after EH is directly dependent on transhepatic flow and remnant liver volume^8–11^. Portal vein flow (PVF), hepatic artery flow (HAF), and portal vein pressure (PVP) have been described as the main critical parameters for the development of SFSF^12,13^. Following EH, the ratio of HAF to the remnant liver weight (HAF/100 g) decreases, while PVF/100 g and PVP increase, resulting in various pathologic consequences that lead to SFSF^10,14–16^. Troisi et al. recommended a flow of 250 ml/min/100 g as the upper limit for PVF to prevent SFSF syndrome^9,17^. Thus, vascular modulation, which is able to decrease PVF/100 g and PVP, as well as increase HAF/100 g following EH, may prevent the occurrence of SFSF^18–21^. The aim of this study was to determine whether a portocaval shunt (PCS) can correct the unwanted changes that occur in transhepatic flow following EH.

## Results

Forty female Landrace pigs aged between 10 and 12 weeks and weighing between 29 and 34 kg (mean 31 ± 2.7 kg) were included in this study. There was no significant difference between the groups with regard to the age or weight of the animals.

### General monitoring

Hemodynamic variables remained stable in all animals for the full duration of the experiment. The MAP, CVP, and HR showed maximum changes of 5 mmHg, 1 mmHg, and 14 beats/min before and after each procedure, respectively, which were not statistically significant. The mean blood loss volume was 75 ± 20 ml during the procedures.

### Portal vein flow (PVF)

**Figure 1:**
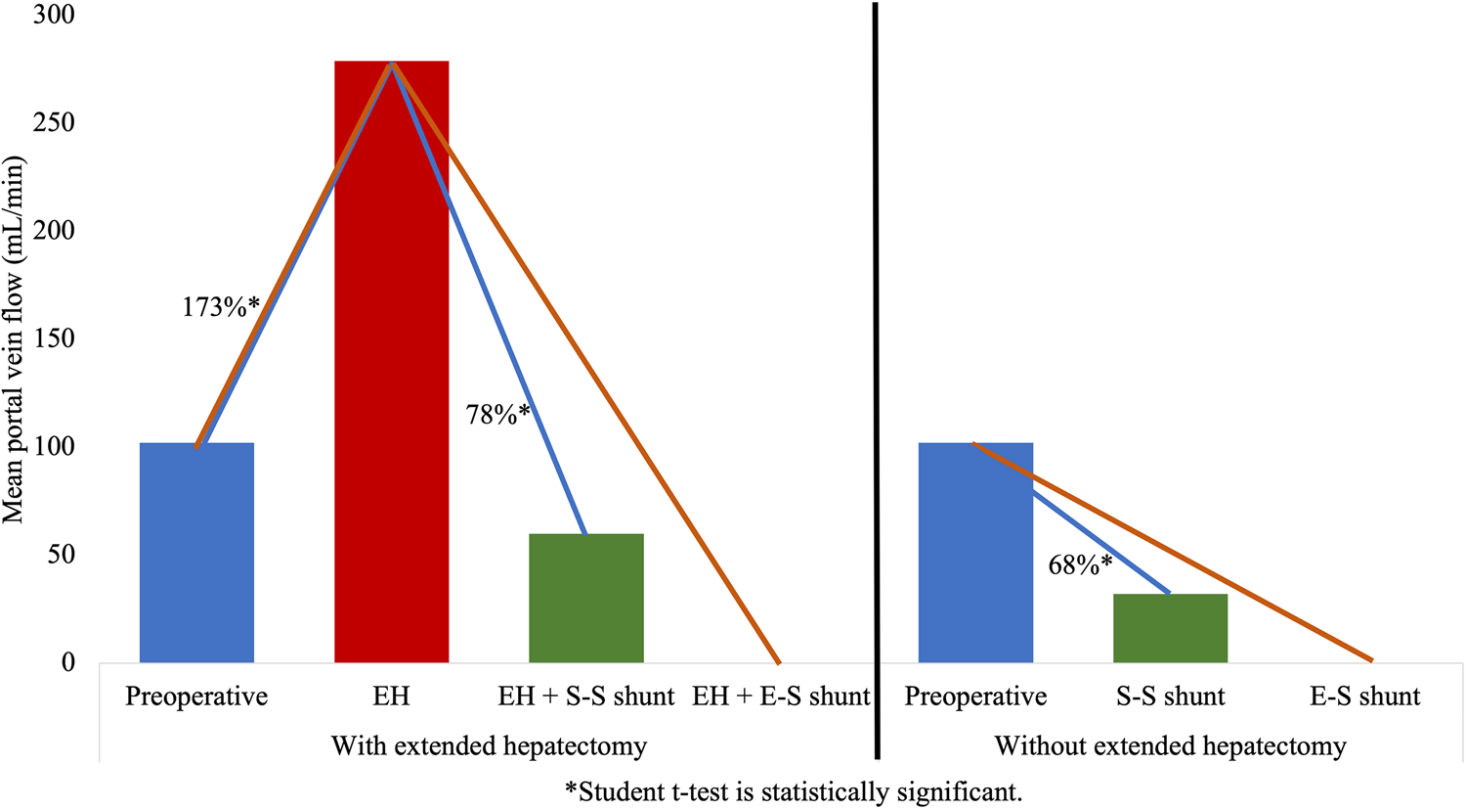
PVF changes with and without EH in three steps without PCS, with a side-to-side portocaval shunt (S–S PCS) and with an end-to-side portocaval shunt (E–S PCS). (1) EH increases the PVF by 173%; (2) S–S PCS reduces the PVF following EH by 78%; (3) S–S PCS reduces the PVF without EH by 68%; (4) S–S PCS following EH corrects the PVF close to the normal value/100 g.

After EH, the PVF/100 g of remnant liver increased from 102.11 ± 9.09 to 279 ± 36.44 ml/min/100 g (173%, p < 0.001; Number 1, Fig. 1). This increased flow following EH was reduced to 60 ± 12.40 ml/min/100 g remnant liver (78%, p < 0.001) through side-to-side PCS (Number 2, Fig. 1). Without EH, side-to-side PCS reduced the PVF from 102.11 ± 9.09 to 32.04 ± 5.46 ml/min/100 g (68%, p < 0.001; Number 3, Fig. 1), which is less than the effect of PCS with EH (p = 0.142; Number 3 < Number 2). The side-to-side PCS corrected the post EH PVF changes so that close to a normal PVF value was reached (Number 4, Fig. 1). The end-to-side PCS reduced the PVF to zero both in the with and without EH groups, since there was no flow in the portal vein following the placement of this shunt (Fig. 1). Changes in PVF following each step are summarized in Table 1.

**Table 1:** The PVF, HAF, and PVP changes (percentage) following side-to-side and end-to-side PCS with and without EH.

	EH* (%)	p	EH + side-to-side PCS** (%)	p	No EH + side-to-side PCS* (%)	p	EH + end-to-side PCS** (%)	p	No EH + end-to-side PCS* (%)	p
PVF (ml/min/100 g)	173 ↑	< 0.001	78 ↓	< 0.001	68 ↓	< 0.001	–	–	–	–
HAF (ml/min/100 g)	22 ↓	0.819	8 ↑	0.555	20 ↑	0.272	35 ↑	0.512	42 ↑	0.075
PVP (mmHg)	68 ↑	< 0.001	38 ↓	0.001	12 ↓	0.237	48 ↓	< 0.001	21 ↓	0.041

*Compared to the normal liver values.

**Compared to the values after EH.

### Hepatic artery flow (HAF)

**Figure 2:**
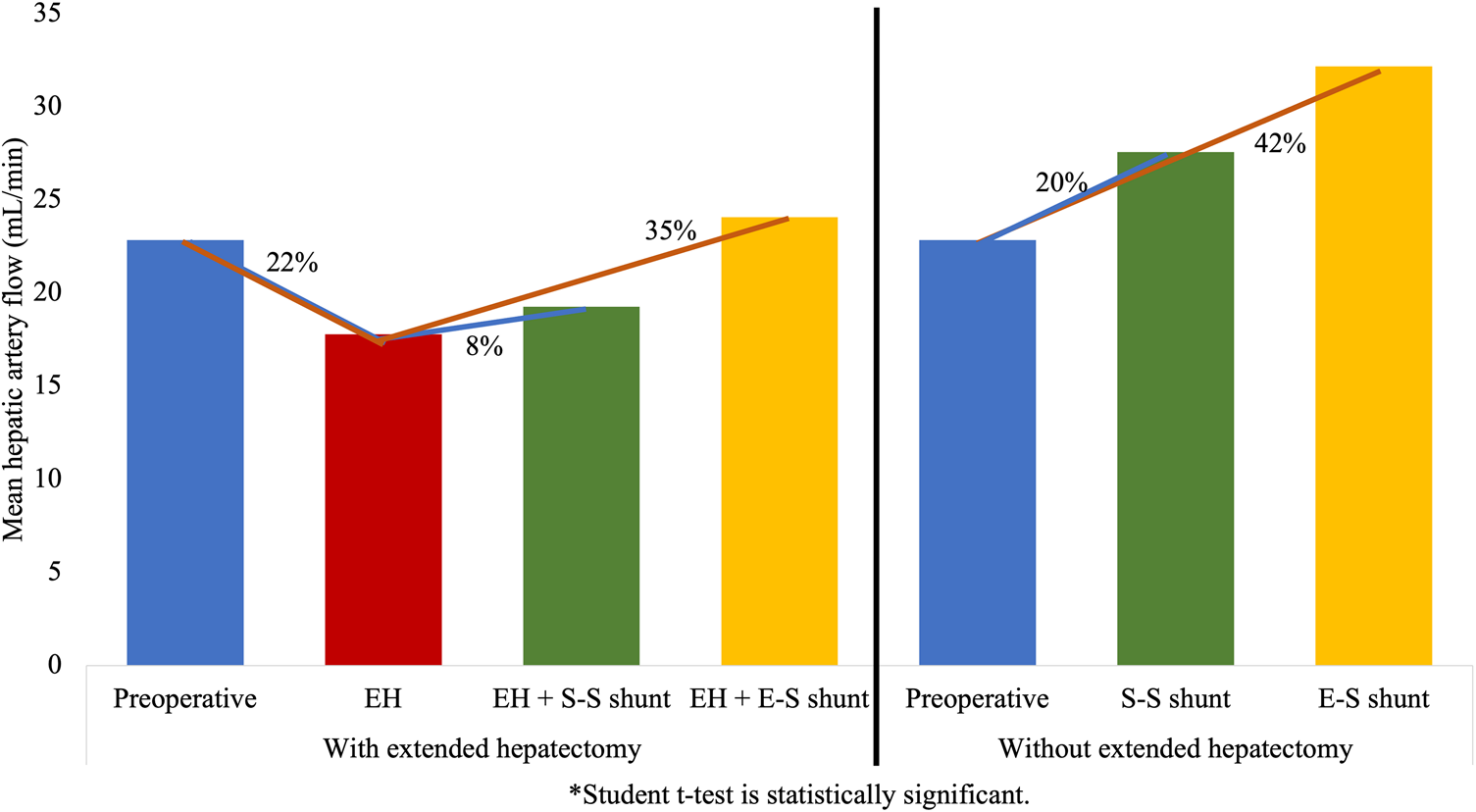
HAF changes with and without EH in three steps without PCS, with side-to-side portocaval shunt (S–S PCS) and with end-to-side portocaval shunt (E–S PCS). (1) EH (75%) reduces the HAF/100 g by 22%; (2) S–S PCS increases the reduced HAF/100 g following EH by 8%; (3) S–S PCS increases the HAF/100 g without EH (20%) more than with EH; (4) The HAF/100 g following EH with an E–S PCS is even higher than the normal HAF/100 g.

EH reduced the HAF/100 g from 22.85 ± 2.62 to 17.77 ± 2.8 ml/min/100 g (22%, p = 0.819; Number 1, Fig. 2). The side-to-side PCS following EH minimally increased the reduced HAF to 19.25 ± 2.9 ml/min/100 g (8%, p = 0.555; Number 2, Fig. 2). Without EH, the side-to-side PCS increased the HAF/100 g from 22.85 ± 2.62 to 27.57 ± 2.9 ml/min/100 g (20%, p = 0.272; Number 3, Fig. 2), which is more than the effect of PCS on HAF following EH (Number 3 > Number 2). The end-to-side PCS increased the HAF/100 g following EH from 17.77 ± 2.8 to 24.07 ± 2.08 ml/min/100 g (35%, p = 0.512) and without EH from 22.85 ± 2.62 to 32.17 ± 2.7 ml/min/100 g (42%, p = 0.041). The HAF/100 g following EH with an end-to-side PCS was even higher than the normal HAF/100 g (Number 4, Fig. 2). The changes in HAF following each step are summarized in Table 1.

### Portal vein pressure (PVP)

**Figure 3:**
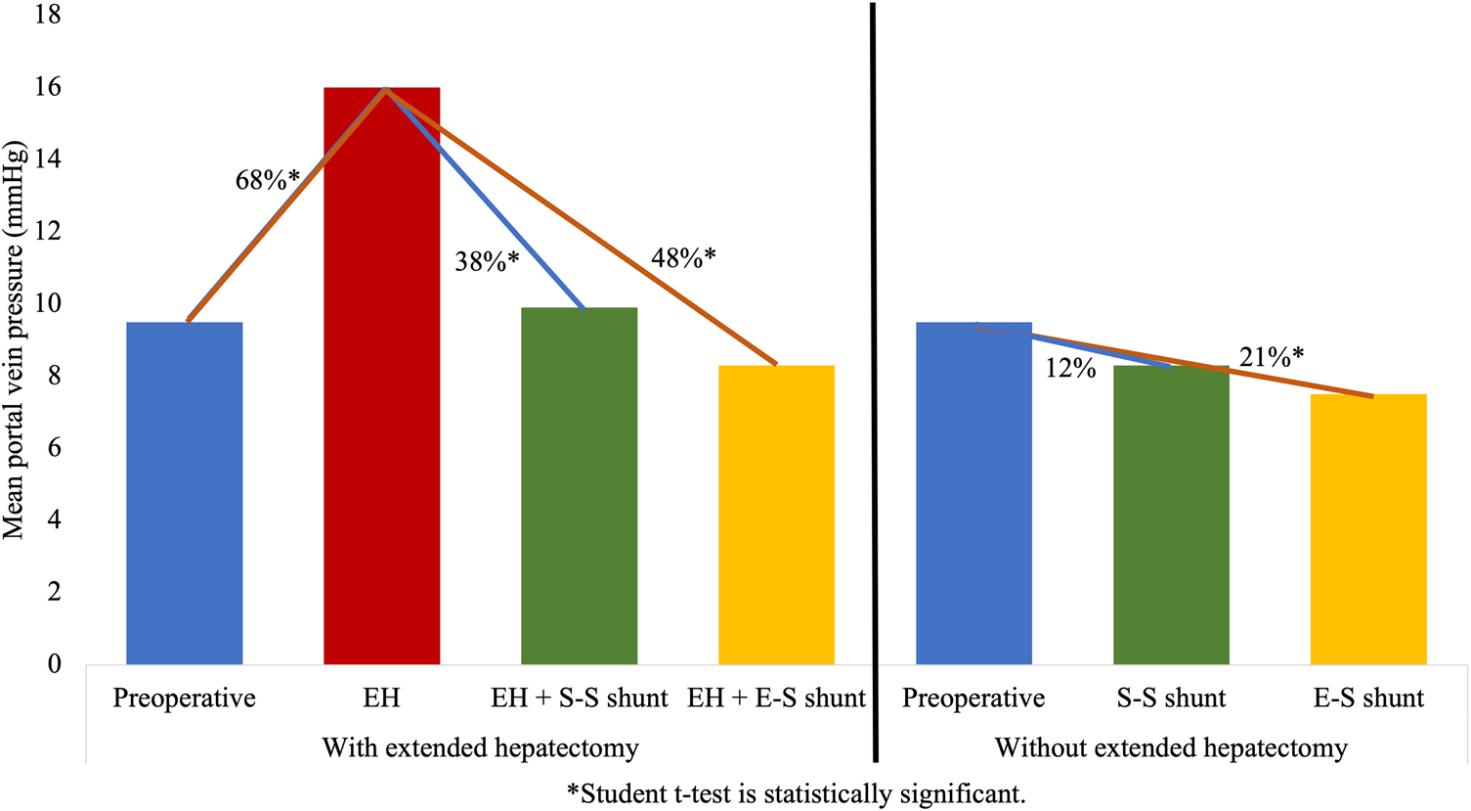
PVP changes with and without EH in three steps without PCS, with a side-to-side portocaval shunt (S–S PCS) and with an end-to-side portocaval shunt (E–S PCS). (1) EH increases the PVP by 68%; (2) S–S PCS reduces the PVP following EH by 38%; (3) S–S PCS reduces the PVP without EH by 12%; (4) E–S PCS reduces the PVP following EH by 48%.

EH increased the PVP from 9.5 ± 0.67 to 16 ± 1.29 mmHg (68%, p < 0.001; Number 1, Fig. 3). The side-to-side PCS following EH reduced the PVP from 16 ± 1.29 to 9.9 ± 0.66 mmHg (38%, p = 0.001; Number 2, Fig. 3). The PVP following EH with side-to-side PCS was close to the normal value. Without EH, the PVP decreased from 9.5 ± 0.67 to 8.3 ± 0.71 mmHg (12%, p = 0.237) as a result of side-to-side PCS (Number 3, Fig. 3). The end-to-side PCS reduced PVP following EH from 16 ± 1.29 to 8.3 ± 1.04 mmHg (48%, p < 0.001) and without EH from 9.5 ± 0.67 to 7.5 ± 0.81 mmHg (21%, p = 0.075). PVP did not significantly change as a result of PCS without EH. The side-to-side PCS after EH reduced PVP up to 80% the values after EH, whereas the end-to-side method reduced the pressure 10 percent more than the side-to-side method (48% vs. 38%; Number 4 vs. Number 2, Fig. 3). The changes in PVP following each step are summarized in Table 1.

## Discussion

Despite the new developments in the field of liver surgery in recent decades, many patients are unable to undergo therapeutic liver resection due to the risk of SFSF syndrome^22^. Posthepatectomy liver failure remains one of the most serious complications of major liver resection and occurs in up to 10% of cases^22,23^. Several studies have reported posthepatectomy liver failure as a significant cause of morbidity and mortality^7^. Considering the important role of transhepatic flow in this regard^24^ as well as its unwanted changes following EH^25^, it can be hypothesized that by a correction of unwanted flow changes following EH, SFSF syndrome may be prevented. It has been shown that reducing the PVF by clamping the splenic artery and performing splenectomy^26^ in small for size grafts following partial liver transplantation can prevent the consequences of SFSF^19,27–29^. PCS, which also reduces PVF, could show similar results in partial liver transplantation^30,31^. The effect of PCS on portal vein decompression has been deemed an important factor in preventing progressive necrosis and ultimately fatal liver failure following partial liver transplantation^32^. While some experimental studies have investigated the role of PCS in rat models following EH^33–35^, the feasibility and the effect of PCS following EH in correction of transhepatic flow, including its effect on hepatic artery flow, have never been systematically studied in human or large animal models. Due to the anatomical and physiological similarities between pigs and humans^36,37^, pigs are optimal models for evaluating the long-term effect of PCS following EH. However, evaluating the feasibility and effectiveness of PCS following EH in a short-term follow-up is one of the ethical and scientific prerequisites.

To systematically evaluate the immediate effect of PCS, we compared the transhepatic flow without PCS and with side-to-side and end-to-side PCS in livers without EH as well as following EH. Our study shows that following EH, the PVF and PVP increase, whereas HAF decreases significantly per 100 g of remnant liver. Moreover, we showed that the PCS, either side-to-side or end-to-side, can correct these variations by reducing the PVF and PVP and increasing the HAF. Following EH with side-to-side PCS, the PVF and PVP were even close to the normal values observed in an intact liver (without resection). This correction was also seen in HAF following EH through end-to-side PCS. As mentioned in our previous study, it is important to compare the results proportional to the remnant liver volume^25^. In the clinical setting and following EH, the remnant liver is exposed to increased PVF and PVP^24^. This leads to centrilobular arterial hypoperfusion and sinusoidal damage and finally to the occurrence of SFSF syndrome with a high rate of postoperative morbidity and mortality^32,38,39^. Following EH, the liver attempts to regenerate itself to be able to provide the needed function for the whole body mass^40^. It has been shown that following EH, the HAF remains constant^41^. It is also known that the liver will automatically compensate for a reduction in PVF by increasing the HAF to hold the transhepatic flow unchanged. This phenomenon is called the hepatic artery buffer response^42^. Therefore, performing a PCS not only correct the unwanted changes in PVF and PVP but can also improve the HAF. Our results could show and confirm this pattern following EH in a porcine model with a short-term evaluation.

PCS can be performed either side-to-side or end-to-side. Based on our results, the side-to-side PCS corrects the PVF and PVP changes following EH up to the normal values in an intact liver (without resection). The end-to-side PCS could show better results in regard to the HAF. This is because the liver loses the whole PVF following an end-to-side PCS and attempts to compensate for this variation by increasing the HAF. In the clinical setting, side-to-side PCS is more practical than end-to-side PCS. Redirecting the whole PVF through an end-to-side PCS can result in high-stage hepatic encephalopathy as well as right heart failure^43,44^. However, end-to-side PCS can be an option in few cases with chronic portal vein obstruction. Although short-term follow-up results are necessary and form a basis for further long-term studies, the absence of a long-term follow-up is admittedly a limitation of this study.

## Conclusion

Hepatic inflow modulation by PCS following EH is feasible. PCS following EH immediately reduces and corrects the increased PVF and PVP and increases the decreased HAF. Based on these findings and since the changes in HAF, PVF, and PVP are considered triggers of SFSF syndrome, further long-term experimental and clinical studies must be performed to evaluate the effectiveness of PCS in preventing SFSF syndrome following EH.

## Methods

### Study design

**Figure 4:**
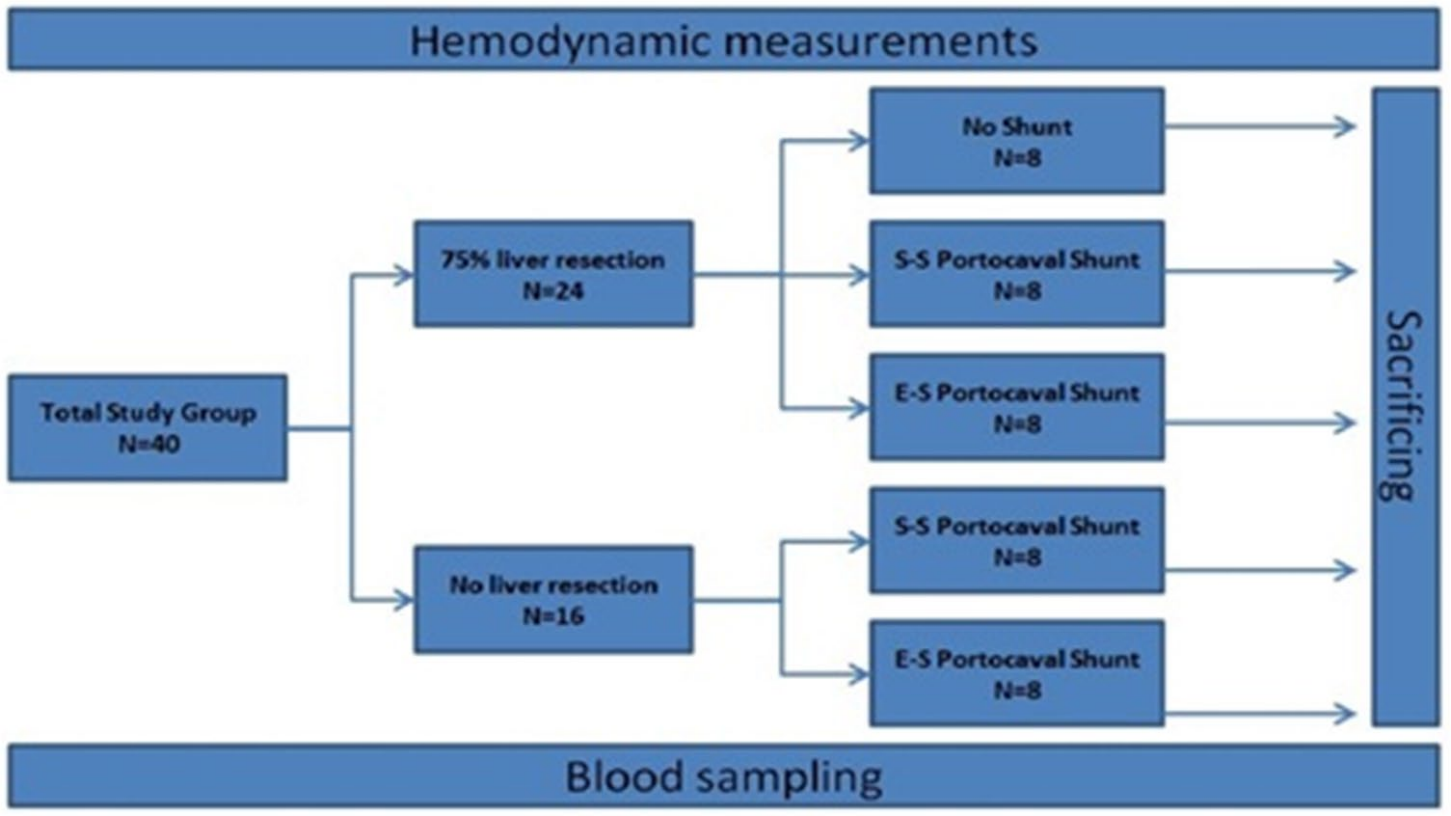
Study design.

The study has been reported in accordance with the ARRIVE guidelines (Animals in Research: Reporting In Vivo Experiments)^45^. This experimental study was conducted on forty female Landrace pigs. The animals were divided into two main groups: (A) EH (75%) and (B) no EH. Group A was further divided into 3 subgroups: (A1) EH without PCS; (A2) EH with side-to-side PCS; and (A3) EH with end-to-side PCS. Group B was divided into 2 subgroups: (B1) side-to-side PCS without EH and (B2) end-to-side PCS without EH (Fig. 4). Each group was made up of 8 animals. HAF, PVF, and PVP were measured in each animal before and after the surgical procedure. The baseline data of the animals before the surgery were considered representative of the group without EH and without PCS.

### Anesthesia, hemodynamics, hepatic inflow and pressure monitoring

After a 12-h fasting time with free access to water, the pigs were anesthetized following our standardized narcotic protocol. Two catheters were placed in the carotid artery and internal jugular vein to monitor MAP and CVP, respectively. The heart rate, body temperature, and blood oxygen saturation were monitored during the entire procedure^37^. A Lohmeier M111 device (Lohmeier Medical GmbH, Munich, Germany) was used to monitor PVP, HAF, and PVF, as described in our previous study^25^. After performing any intervention, we monitored the PVF, HAF, and PVP in each study group until the values reached a stable level. The HAF and PVF are reported in ml per 100 g remnant liver.

### Surgical procedure

**Figure 5:**
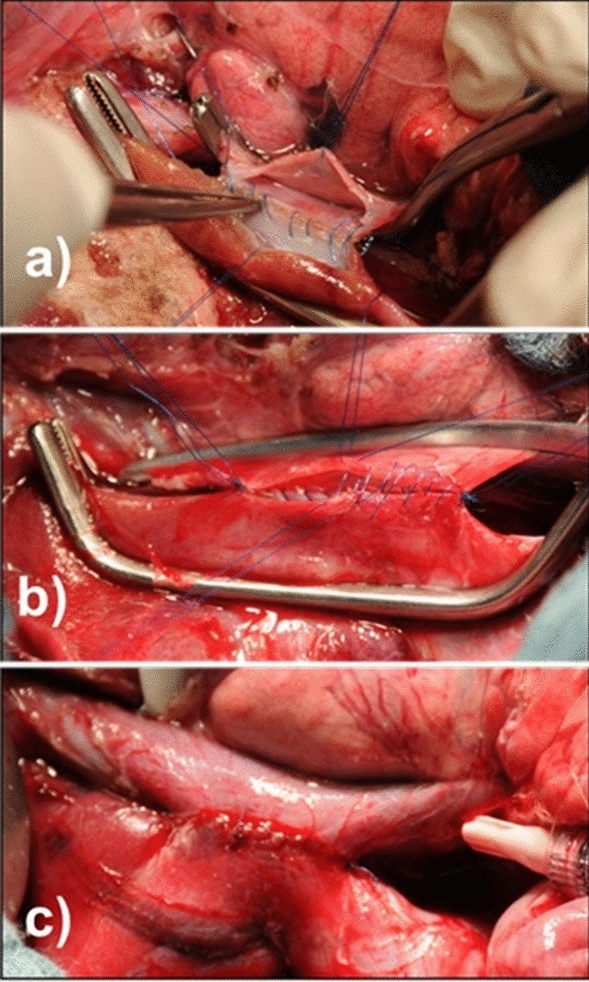
Side-to-side portocaval shunt (S–S PCS): (**a**) stitching the posterior wall of the anastomosis; (**b**) stitching the anterior wall of the anastomosis; (**c**) the complete anastomosis.

**Figure 6:**
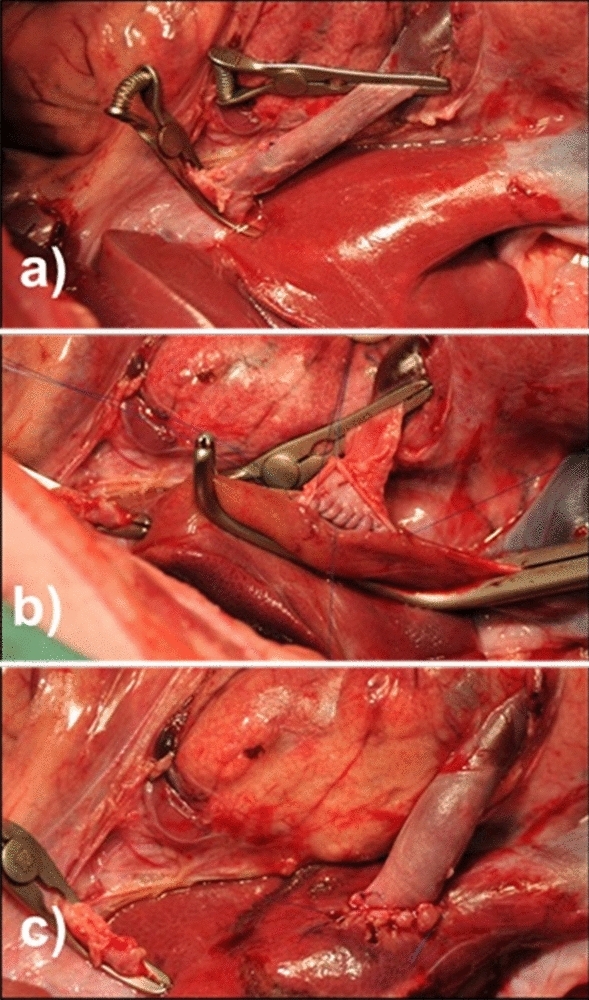
End-to-side portocaval shunt (E–S PCS): (**a**) cross clamping of the portal vein; (**b**) stitching of the posterior wall of the anastomosis; (**c**) complete anastomosis.

The resection procedure was conducted in accordance with our standardized stapler hepatectomy method^46^. After EH, complete hemostasis was achieved by electrocoagulation and hand suturing. To perform the PCS, the portal vein and vena cava were prepared and released from the surrounding tissues in the potential anastomosis place. Both veins were cross-clamped after heparinization with 30 IU/kg. For side-to-side PCS placement, a portal vein-caliber size anastomosis was performed between the right lateral side of the portal vein and medial side of the vena cava using Prolene 5–0 (PROLENE® Ethicon, Norderstedt, Germany) in a continuous technique (Fig. 5). To place the end-to-side PCS, a portal vein-caliber size venotomy was performed on the vena cava after the portal vein in the liver hilum was cross-clamped and dissected. Anastomosis was performed continuously with the end-to-side technique (Fig. 6). At the end of the experiment, the remnant liver was resected and weighed to determine the hepatic hemodynamic values related to the remnant liver weight (per 100 g).

### Animal rights

The study protocol was approved by the German Committee for Animal Care, Karlsruhe, Germany (AZ: 35-9185.81/G-45/12). In accordance with the institutional guidelines established for the Animal Care Facility at the University of Heidelberg, all animals received human care during the experiment. Once the surgical procedures were completed and the hemodynamic measurements were obtained, the animals were euthanized by an intravenous injection of potassium chloride (2 mmol/kg) under deep anesthesia.

### Statistical analysis

The statistical analysis was performed using SPSS 22 (IBM Corp. Released 2013. IBM SPSS Statistics for Windows. Armonk, NY: IBM Corp). All measured values are expressed as the mean ± standard deviation (SD), and the changes in the values are graphically represented. The differences in mean hemodynamic measurements between study groups were tested using independent samples t tests. A p value less than 0.05 was considered to be statistically significant in all tests.

### Institutional protocol number

The study protocol was approved by the German Committee for Animal Care, Karlsruhe, Germany (AZ: 35–9185.81/G-45/12).

## Abbreviations

EH: Extended hepatectomy
HAF: Hepatic artery flow
PVF: Portal vein flow
PVP: Portal vein pressure
SFSF: Small for size and flow
PCS: Portocaval shunt
